# LGE-PSIR is an independent predictor of mortality in cardiac amyloidosis: a 250 patient prospective study

**DOI:** 10.1186/1532-429X-17-S1-O27

**Published:** 2015-02-03

**Authors:** Marianna Fontana, Silvia Pica, Patricia Reant, Amna Abdel-Gadir, Thomas A Treibel, Sanjay M Banypersad, Viviana Maestrini, Heerajnarain Bulluck, Thirusha L Lane, Helen Lachmann, Carol J Whelan, Ashutosh Wechalekar, Charlotte Manisty, Anna S Herrey, Peter Kellman, Philip N Hawkins, James Moon

**Affiliations:** 1 National Amyloidosis Centre, London, UK; 2 Heart Hospital, London, UK; 3 National Institutes of Health / NHLBI, Bethesda, MD, USA; 4 Heart Hospital, London, UK

## Background

CMR with late gadolinium enhancement technique (LGE) is a candidate reference standard for non invasive diagnosis of light chain and transthyretin cardiac amyloidosis (AL, ATTR). However, the nulling is difficult and conflicting results on morphology and LGE have been reported in small retrospective studies. We hypothesize that morphology and LGE can guide differential diagnosis and predict survival in AL and ATTR amyloidosis.

## Methods

250 patients 119 with AL, 131 with ATTR (9 of which were mutations carriers) underwent CMR with standard cine imaging, LGE (with magnitude-reconstructed images, magnitude-IR, and phase sensitive inversion recovery reconstruction, PSIR) and T1 mapping with Extracellular Volume (ECV) measurement.

## Results

### Morphology AL vs ATTR

Compared to AL, ATTR had more LVH, more asymmetry, more LV impairment and more RVH (for all p<0.05).

### PSIR superior to Magnitude-IR

**Figure 1:**
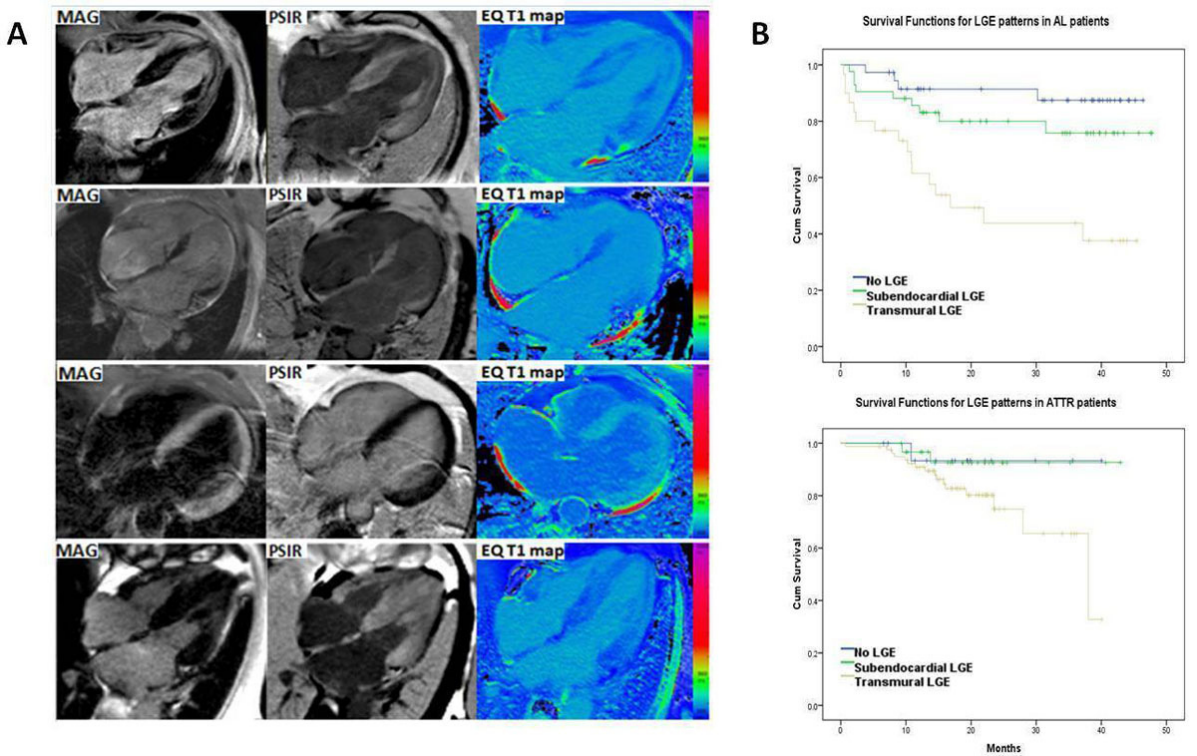
**Panel A.** Late gadolinium enhancement (LGE) with magnitude IR (left panels). LGE with phase sensitive inversion recovery reconstruction (PSIR) (middle) and post contraset ShMOLLI T1 maps (right panels). There is concordance between areas of low T1 (blue) in the Tw maps and areas of LGE in the PSIR image but discordance with the magnitude image. **Panel B:** Kaplan Meier curves for PSIR late gadolinium enhancement patters in AL (upper panel) and ATTR patients (Lower panel).

Using the ECV as truth standard (LGE should have lower T1 than nulled myocardium), magnitude-IR had incorrect initial regional or global nulling in 43% (i.e. myocardium with the highest ECV displayed as nulled). PSIR was always right and displayed areas of greatest interstitial expansion as bright (P<0.0001), even when pan-myocardial (Figure 1A).

### PSIR-LGE baseline

PSIR LGE could be divided into three patterns (AL vs ATTR respectively): none (34% vs 13%, p<0.05), subendocardial (39% vs 24%, p<0.05) and transmural (27% vs 63%, p<0.0001). PSIR-LGE tracks increasing amyloid burden measured as ECV (and DPD grade in ATTR), p<0.0001.

### PSIR-LGE mortality

During an average follow-up of 22 months, 52 deaths occurred, 32 in AL and 20 in ATTR. Transmural LGE predicted death in AL (HR 6.6, 95% CI: 2.1-19.7, P=0.001) (Figure 1B) and remained independent after adjusting for NT-proBNP, EF, E/E' (HR = 3.6, 95% CI: 1.1-11.8). In ATTR, all cardiac deaths happened in transmural patients except for one that was classified discordantly by the two observers (subendocardial and transmural).

## Conclusions

AL and ATTR have different morphologies. PSIR LGE completely solves the LGE nulling problem in amyloid. Using PSIR LGE permits 3 patterns of LGE to distinguished. A transmural PSIR LGE pattern in amyloid is an independent predictor of death.

## Funding

The project is supported by a doctoral research fellowship by the British Heart Foundation (FS/12/56/29723).

